# Near-Infrared Radiation-Based Mild Photohyperthermia Therapy of Non-Melanoma Skin Cancer with PEGylated Reduced Nanographene Oxide

**DOI:** 10.3390/polym12081840

**Published:** 2020-08-17

**Authors:** Raquel Costa-Almeida, Diana Bogas, José R. Fernandes, Licínia Timochenco, Filipa A. L. S. Silva, João Meneses, Inês C. Gonçalves, Fernão D. Magalhães, Artur M. Pinto

**Affiliations:** 1 i3S—Instituto de Investigação e Inovação em Saúde, Universidade do Porto, 4200-180 Porto, Portugal; rcalmeida@i3s.up.pt (R.C.-A.); flsilva@i3s.up.pt (F.A.L.S.S.); icastro@ineb.up.pt (I.C.G.); 2 INEB—Instituto de Engenharia Biomédica, Universidade do Porto, Rua Alfredo Allen, 208, 4200-180 Porto, Portugal; 3 LEPABE, Faculdade de Engenharia, Universidade do Porto, 4200-180 Porto, Portugal; dianabogas@fe.up.pt (D.B.); up201809122@fe.up.pt (L.T.); up201503132@fe.up.pt (J.M.); fdmagalh@fe.up.pt (F.D.M.); 4 CQVR—Centro de Química Vila Real, Departamento de Física, ECT, Universidade de Trás-os-Montes e Alto Douro, 5001-801 Vila Real, Portugal; jraf@utad.pt

**Keywords:** graphene, light emitting diode, phototherapy, polyethylene glycol, thermal reduction

## Abstract

Using a one-step thermal reduction and non-covalent chemical functionalization process, PEGylated reduced nanographene oxide (rGOn-PEG) was produced from nanographene oxide (GOn) and characterized in terms of particle size, dispersion stability, chemistry, and photothermal properties, in view of its use for photothermal therapy (PTT) of non-melanoma skin cancer. GOn infrared spectrum presented more intense bands assigned to oxygen containing functional groups than observed for rGOn-PEG. GOn C/O ratio decreased more than 50% comparing with rGOn-PEG and nitrogen was present in the latter (N *at* % = 20.6) due to introduction of PEG-NH_2_. Thermogravimetric analysis allowed estimating the amount of PEG in rGOn-PEG to be of about 56.1%. Simultaneous reduction and PEGylation increased the lateral dimensions from 287 ± 139 nm to 521 ± 397 nm, as observed by transmission electron microscopy and dynamic light scattering. rGOn-PEG exhibited ≈13-fold higher absorbance in the near-infrared radiation (NIR) region, as compared to unmodified GOn. Low power (150 mW cm^−2^) NIR irradiation using LEDs resulted in rGOn-PEG heating up to 47 °C, which is within the mild PTT temperature range. PEGylation strongly enhanced the dispersibility of rGOn in physiological media (phosphate buffered saline, fetal bovine serum, and cell culture medium) and also improved the biocompatibility of rGOn-PEG, in comparison to GOn (25–250 μg mL^−1^). After a single NIR LED irradiation treatment of 30 min, a decrease of ≈38% in A-431 cells viability was observed for rGOn-PEG (250 μg mL^−1^). Together, our results demonstrate the potential of irradiating rGOn-PEG using lower energy, cheaper, smaller, and safer LEDs, as alternative to high power lasers, for NIR mild hyperthermia therapy of cancer, namely non-melanoma skin cancer.

## 1. Introduction

Non-melanoma skin cancer (NMSC) has been reported as one of the most common types of cancer worldwide, with an estimation of over 3 million diagnoses each year in the USA [1]. Despite including different malignancies, basal cell and squamous cell carcinomas are the most frequent types of NMSC [1,2,3]. Depending on disease risk level, surgical excision or radiotherapy are the standard treatment options. Severe cases of high-risk primary, recurrent, or metastatic NMSC frequently require multimodal therapy [1,3,4,5]. However, functional and cosmetic outcomes are still variable, requiring novel treatment strategies.

Photothermal therapy (PTT) is being increasingly explored as an alternative non-invasive cancer treatment. It relies on tumor irradiation with a near-infrared (NIR) laser, either topically or interstitially through an optical fiber, leading to light energy conversion into heat, ultimately resulting in tumor ablation [6]. Two different mechanisms can be broadly identified. Particularly, hyperthermia results from mild temperature increases leading to the activation of cellular apoptotic pathways, whereas tumor ablation is achieved with rapid temperature increases (> 50 °C) [7]. Nanomaterials hold potential as photothermal absorbers to enhance PTT selectivity within the target tumor tissue toward achieving therapeutic temperatures (> 41 °C) using less total light energy, minimizing damage to the healthy surrounding tissue [6].

Over the past decade, graphene-based materials (GBM) have been widely explored for a huge number of applications, including energy, aerospace, biomedicine, and health [8,9,10,11,12,13,14,15]. Recently, GBM emerged as potential PTT-based cancer therapy platforms owing to their high NIR absorption [16,17,18]. Similarly to graphene-based materials, other 2D nanomaterials are being increasingly explored for PTT cancer treatment, including black phosphorus (BP) and transition metal dichalcogenides (TMDs), among others [17]. However, BP is highly sensitive to water and oxygen [19] and frequently requires the combination with gold nanoparticles to achieve NIR photothermal transduction efficiency [20,21]. In turn, TMDs are gaining interest, but low-yield and low-quality exfoliation methods still pose enormous challenges to their application in biomedicine [22,23].

Graphene oxide (GO) has been receiving increased attention in the field of biomedicine due to its surface chemistry with abundant oxygen reactive functional groups that enables a wide range of chemical modifications and bioconjugation approaches [13,24,25,26]. We have previously shown that oxidation of graphene nanoplatelets resulted in enhanced in vitro biocompatibility of these 2D nanomaterials when cultured with human dermal fibroblasts at concentrations up to 100 µg mL^−1^ [27]. However, while improving water dispersibility and biocompatibility, oxidation of graphene into GO results in diminished electronical and optical conductivity [24,28]. Chemical, thermal, and electro/photochemical reduction methods are widely investigated to restore the aromatic structure of graphene and obtain reduced GO with high NIR absorption capacity and biocompatibility for applications in human health [13]. Over the years, green reduction combined with chemical functionalization methods using biocompatible polymers, such as polyethylene glycol (PEG), to act as surfactants have been reported [29,30,31,32,33,34]. Such methods yield NIR-absorbing photothermal agents that can strongly absorb NIR light and convert it into cytotoxic temperature increases for local hyperthermia.

In the present work, we envisioned to develop a graphene-based PTT agent to be explored for low power NIR-induced photohyperthermia therapy with prospective application in the treatment of NMSC. To achieve this goal, key requisites were considered: (1) a nano-sized material to enhance skin permeation and retention [35,36]; (2) strong NIR absorption capacity to enable low power NIR-triggered hyperthermia; and (3) biocompatibility in the absence of NIR irradiation. Building up on a previously described protocol for single step thermal reduction and PEGylation of graphene oxide [37], we prepared non-covalently functionalized nanosized reduced graphene oxide (rGOn-PEG) and characterized in detail the impact of this process on the physicochemical properties of developed materials through multiple complementary techniques. Herein, we obtained a stable dispersion of rGOn-PEG through a ‘water-only’ reduction protocol. This study enabled us to identify the main chemical features and quantify the chemical modification obtained through such a green and facile method. We further explored the developed material as a NIR-absorbing agent for PTT treatment using low power NIR light source to achieve a hyperthermia effect, envisioning application in the treatment of superficial non-melanoma skin cancers, which constitutes a novel approach.

## 2. Materials and Methods

### 2.1. Synthesis of Nanographene Oxide (GOn)

Graphene oxide was prepared from graphite powder (size ≤ 20 µm, Sigma Aldrich, St. Louis, MO, USA) by oxidation using the modified Hummer’s method, as described before [27,38]. Briefly, a mixture of 320 mL of sulfuric acid (H_2_SO_4_, VWR, Darmstadt, Germany) and 80 mL of phosphoric acid (H_3_PO_4_, Chem-Lab, Zedelgem, Belgium) was added to 8 g of graphite while stirring, and the solution was cooled using an ice bath. Then, 48 g of potassium permanganate (KMnO_4_, JMGS, Odivelas, Portugal) were added gradually and the solution was heated to 35 °C and stirred for 2 h. Subsequently, 1200 mL of H_2_O were slowly added under stirring and with temperature control using an ice bath. Finally, hydrogen peroxide (H_2_O_2_, VWR, Darmstadt, Germany) was added to stop the reaction. After overnight resting, the solution was decanted to separate the solid phase from the acidic solution, centrifuged at 4000 rpm for 20 min, and redispersed in distilled water. The process was repeated until achieving a neutral pH in the supernatant. In order to produce smaller GO flakes, the pellet recovered in the previous step was re-dispersed in distilled water and placed in an ultrasonic bath (Ovan ATM40-3LCD) for 4 h. The sonication was followed by a centrifugation step at 13,000 rpm for 30 min, allowing the separation of two different phases. The upper phase corresponds to nanographene oxide (GOn) particles and was recovered for further use.

### 2.2. One-Step Reduction and PEGylation of GOn

Reduced nanographene oxide (rGOn) was produced and functionalized through a single step process, as previously described [37]. For this purpose, 10 mL of GOn (500 µg mL^−1^) were mixed with 50 mg of poly(ethylene glycol) bis(amine) (PEG-NH_2_, average *M*_n_ = 3350, Sigma, St. Louis, MO, USA). The mixture was then homogenized using an ultrasonic bath (Ovan ATM40-3LCD) for 10 min. Finally, the mixture was left in a water bath for 24 h at 90 °C. In order to remove unstable aggregates and excess PEG-NH_2_, rGOn-PEG was washed four times with deionized water and centrifuged at 13,000 rpm for 10 min. After the washing step, the pellet was redispersed in water to obtain purified rGOn-PEG suspensions. As controls, GOn dispersions incubated with PEG were maintained at room temperature for 24 h; and rGOn was prepared following the same thermal reduction protocol in the absence of PEG.

### 2.3. Physicochemical Characterization of Graphene-Based Materials

#### 2.3.1. Transmission Electron Microscopy

Morphology and lateral dimensions of GOn and rGOn-PEG dispersions were analyzed by transmission electron microscopy (TEM, JEOL JEM 1400 TEM, Tokyo, Japan). Aqueous dispersions were prepared at a concentration of 50 µg mL^−1^. For each sample, 10 µL were deposited on a carbon coated TEM grid and allowed to sediment for 1 min, followed by excess material removal by capillarity using filter paper. Nanomaterial lateral dimensions were measured on several TEM images using ImageJ software. Results are presented as frequency distribution of lateral dimensions from over 100 or 25 particles for GOn or rGOn-PEG, respectively.

#### 2.3.2. Dynamic Light Scattering

Particle size distributions for GOn and rGOn-PEG were determined by dynamic light scattering (DLS, LS230 particle size analyzer, Beckman Coulter, Brea, CA, USA). The materials were dispersed in water at a concentration of 100 µg mL^−1^ prior to the measurements. Data were collected performing two scans of 60 s, including polarization intensity differential scattering and using Fraunhofer’s model. This model assumes spherical shape for particles in suspension. This evaluation of size distributions does not correspond to precise estimations of particle size and must be considered as relative evaluations of deagglomeration of the different materials in water [27].

#### 2.3.3. Zeta Potential Measurements

Prior to zeta potential measurements, GOn and rGOn-PEG dispersions were prepared at a concentration of 50 µg mL^−1^ and pH 7.4. Zeta potentials of aqueous dispersions were determined using a Zetasizer Nano-ZS (Malvern Instruments, Worcestershire, UK) in a disposable Zetasizer cuvette (Malvern Instruments, Worcestershire, UK). Each measurement was performed in triplicate at room temperature and results are reported as mean and standard deviation.

#### 2.3.4. UV/Visible Spectroscopy

Absorption spectra of GOn and rGOn-PEG were acquired using a Lambda 35 UV/vis spectrometer (Perkin-Elmer, Waltham, MA, USA). Samples were transferred to a 160 µL quartz cuvette (Hellma Analytics, Analytica Munich, Germany) with 10 mm light path length and spectra were recorded in the range of 200–850 nm. Measurements were performed at room temperature by averaging three scans with baseline correction based on water as a blank control.

#### 2.3.5. Fourier Transform Infrared (FTIR) Spectroscopy

Infrared spectra of GOn and rGOn-PEG dehydrated samples were recorded using a VERTEX 70 FTIR spectrometer (Bruker, Karlsruhe, Germany) in transmittance mode at room temperature. Samples were measured in ATR mode, with a A225/Q PLATINUM ATR Diamond crystal with single reflection accessory. Spectra were recorded by averaging 64 scans at a resolution of 4 cm^−1^ over the wavenumber range between 4000 and 400 cm^−1^.

#### 2.3.6. X-ray Photoelectron Spectroscopy

X-ray photoelectron spectroscopy (XPS) analysis was performed at CEMUP (Centro de Materiais da Universidade do Porto, Porto, Portugal) using a Kratos Axis Ultra HSA for data acquisition. For analysis, a monochromator Al X-ray source operating at 15 kV (90 W) was used. Survey XPS spectra were acquired with pass energy of 80 eV, 1 eV step size and 200 ms dwell time. High resolution C1s XPS spectra were acquired with pass energy of 40 eV, 0.1 eV step size, and 1000 ms dwell time. Spectra were processed using CasaXPS software (Casa Software Ltd., Teignmouth, UK). The effect of the electric charge was corrected by calibrating all samples to the reference of the carbon peak (284.6 eV).

#### 2.3.7. Thermogravimetric Analysis

Thermogravimetric analysis (TGA) (Netzsh STA 449 F3 Jupiter, Selb, Germany) was used for comparison of different materials weigh loss under a constant temperature increase. Sample amounts ranged from 4 to 4.5 mg. The thermograms were recorded between 30 and 1000 °C at a heating rate of 10 °C min^−1^ under nitrogen flow. Results are presented as percentage (%) of weight loss.

### 2.4. Photothermal Properties of rGOn-PEG

To evaluate the light-to-heat conversion ability of GOn and rGOn-PEG, 150 µL of GOn, and rGOn-PEG dispersions at different concentrations in a range between 25 and 250 µg mL^−1^ and water (used as control) were placed in a 48-well plate. All samples were irradiated with a LED-based source with a peak emission around 810 nm (NIR region) and irradiance of 150 mW cm^−2^. The light-induced temperature change on the samples was monitored during 30 min of irradiation, using a type K thermocouple placed centered and half-height in the suspension. Three replicates were used per condition and results are presented as mean and standard deviation of absolute temperature.

### 2.5. In Vitro Studies

#### 2.5.1. Cell Culture

Biological studies were performed using A-431 human epidermoid carcinoma cells (ATCC, CRL-1555). Cells were cultured in Dulbecco’s modified Eagle’s medium (DMEM, ATCC) supplemented with 10% (*v/v*) fetal bovine serum (Alfagene, Carcavelos, Portugal) and 1% (*v/v*) penicillin/streptomycin (Biowest, Pays De La Loire, France). Cells were maintained in a humidified atmosphere with 5% CO_2_/95% air at 37 °C.

#### 2.5.2. Cytotoxicity Assays

The effect of GOn and rGOn-PEG on cell viability was evaluated using different concentrations in a range between 25 and 250 µg mL^−1^. Cells were seeded in 48-well plates at a density of 4 × 10^4^ cells/well, incubated at 37 °C and 5% CO_2_. Upon sub-confluence (24 h), culture medium was replaced by GOn or rGOn-PEG dispersions in a final volume of 150 µL/well (in complete DMEM) and cells were incubated with the materials for 24 h. Then, cell viability was quantified by resazurin assay. Briefly, material dispersions were removed, cells were washed with PBS and incubated in 10% (*v/v*) resazurin reagent (Sigma-Aldrich, St. Louis, MO, USA) in culture medium at 37 °C and 5% CO_2_ for 4 h. The fluorescence (*λ*_ex/em_ = 530/590 nm) of the supernatant was measured using a micro-plate reader spectrophotometer (Synergy Mx, Bio-Tek Instruments, Winooski, VT, USA). Negative and positive controls for cell viability decrease were performed by incubating A-431 cells with complete DMEM and 10% (*v/v*) dimethyl sulfoxide (DMSO) in complete DMEM, respectively. Data for each sample were normalized to the negative control and results are presented as % of control. All assays were performed in triplicate with six replicates for each condition tested.

#### 2.5.3. Photothermal Irradiation Assays

To evaluate the combined effect of GBM and NIR irradiation, A-431 cells were seeded and incubated with rGOn-PEG dispersions at increasing concentrations (25–250 µg mL^−1^), as described above. After 24 h of incubation with rGOn-PEG, cells were irradiated for 30 min using a LED-based source with peak emission around 810 nm (NIR region) and irradiance of 150 mW cm^−2^. Immediately after irradiation, the medium containing rGOn-PEG dispersions was removed, cells were washed with PBS, and resazurin assay was performed as described above. For this purpose, negative and positive controls were performed using irradiated A-431 cells with complete DMEM and 10% (*v/v*) dimethyl sulfoxide (DMSO) in complete DMEM, respectively. To compare the effects of increasing concentrations of rGOn-PEG in the presence (LED on) or absence (LED off) of NIR irradiation, results are presented as mean relative fluorescence units (*RFU*) and standard deviation. To compare the effects of NIR irradiation, a negative control in the absence of materials was considered for normalization. Additionally, GOn dispersions at the same concentration were included in NIR irradiation assays as non-absorbing materials for comparison. All assays were performed in triplicate with six replicates for each condition tested.

### 2.6. Statistical Analyses

Statistical analyses were performed using GraphPad Prism software (version 8.4.2, San Diego, CA, USA). One-way and two-way analysis of variance (ANOVA) with Tukey tests for multiple comparisons were performed. Differences between experimental groups were considered significant with a confidence interval of 95%, whenever *p <* 0.05.

## 3. Results

### 3.1. GBM Morphological Properties, Particle Size, and Stability

**Figure 1:**
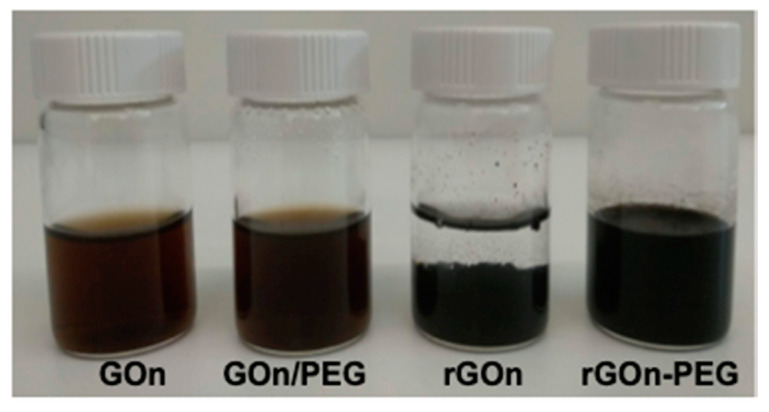
Images of as-prepared GBM dispersions (500 µg mL^−1^) in glass vials for stability evaluation. From left to right: GOn dispersion; GOn/PEG (GOn mixed with PEG without thermal treatment); rGOn dispersion (after thermal treatment without PEG functionalization); rGOn-PEG (simultaneously reduced and non-covalently PEGylated).

Graphene oxide (GO) was prepared from graphite using a modified Hummer’s method, followed by ultrasonication to obtain nanosized GO (GOn). GOn was then reduced and non-covalently PEGylated through a one-step procedure, as previously reported [37]. For this purpose, GOn was mixed with PEG-NH_2_ for 24 h under thermally reducing conditions. Figure 1 shows as-prepared GBM dispersions. GOn presented its typical appearance, as a brownish stable aqueous dispersion. Conjugation of GOn and PEG in aqueous solution at room temperature similarly resulted in a dark brown dispersion (GOn/PEG), as no reducing conditions were present. During thermal reduction of GOn, in absence of PEG, the dispersion progressively changed its color, evidencing the formation of a black precipitate (rGOn). In opposition, non-covalent functionalization of rGOn with PEG (rGOn-PEG) during thermal reduction resulted in a stable aqueous dispersion.

**Figure 2:**
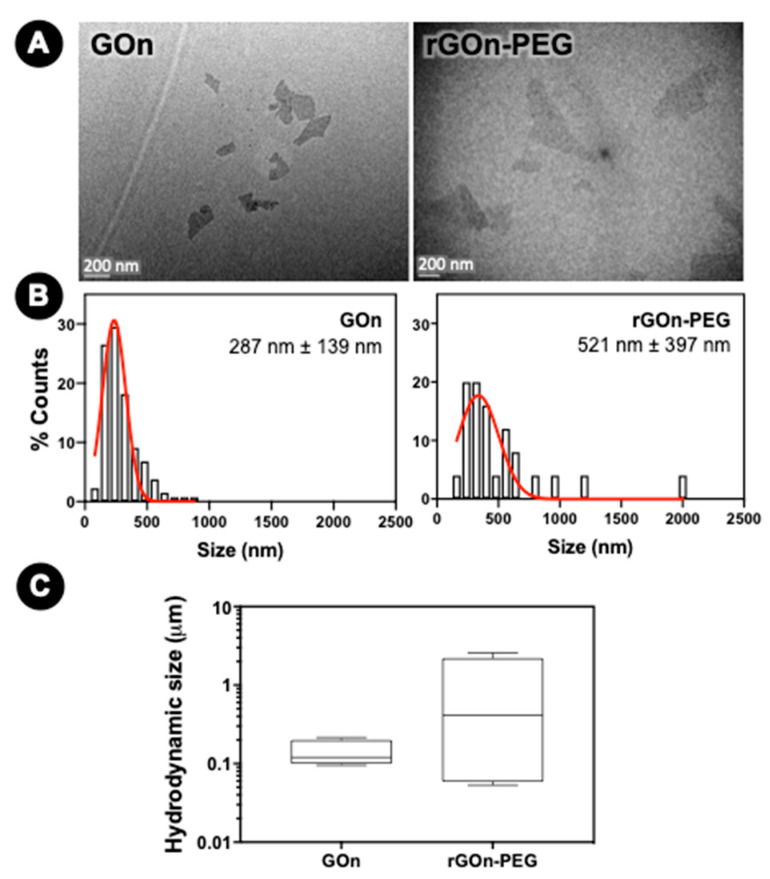
Morphological properties of GOn and rGOn-PEG. (**A**) Representative TEM images of GOn (left) and rGOn-PEG (right) aqueous dispersions and (**B**) respective distribution of particle size and mean and standard deviation, as determined from TEM images. Scale bar, 200 nm. (**C**) Box plot of particle size distributions in volume percentage of GOn and rGOn-PEG dispersed in water at an initial concentration of 250 μg mL^−1^ and determined by light scattering using a Coulter counter.

The morphology of GBM nanosheets was studied using transmission electron microscopy (TEM). Figure 2A shows TEM images of few-hundred-nanometer GOn and rGOn-PEG nanosheets. TEM results are in agreement with previously observed good aqueous dispersibility of GOn and rGOn-PEG, as no agglomerates were observed. Lateral dimensions were determined from TEM image analysis and frequency distribution histograms are shown in Figure 2B. GOn nanosheets were obtained with an average size of 287 nm (minimum and maximum values of 99 nm and 848 nm, respectively, Figure 2A,B). A larger size distribution was observed for rGOn-PEG, which exhibited an average size of 521 nm (minimum and maximum values of 162 nm and 2028 nm, respectively, Figure 2A,B). Similarly, particle size determinations by DLS revealed that GOn nanosheets were constituted by considerably smaller particle sizes than rGOn-PEG (Figure 2C, Figure S1). Figure S1 presents the volume distribution of GOn and rGOn-PEG particle size, whereas Figure 2C corresponds to box plot representations of the same results. For GOn, there were two subpopulations with peaks averaging around 93 nm and 195 nm (Figure S1). From both peaks, the determined average for GOn in general was of 135 nm, while the median was of 120 nm (Figure 2C). The minimum particle size was of 48 nm and the maximum size of 284 nm (Figure 2C, Figure S1). For rGOn-PEG, there were four subpopulations with peaks average at around 64 nm, 214 nm, 545 nm, and 2423 nm (Figure S1). From these peaks, the average was determined to be of 928 nm, while the median value corresponded to 413 nm (Figure 2C). The minimum particle size for rGOn-PEG was of 40 nm, whereas the maximum size was of 3206 nm (Figure 2C, Figure S1).

Both GOn and rGOn-PEG exhibited comparable colloidal stability in aqueous dispersions, according to zeta (*ζ*)-potential measurements (Table 1). GOn displayed a greater negative surface charge (−25.1 ± 0.8 mV) than rGOn-PEG (−10.2 ± 0.3 mV). This suggests the existence of positive amino-ended branches, resulting in a reduced negative electrostatic charge for non-covalently PEGylated rGOn when compared with GOn [33].

### 3.2. Chemical Characterization of GOn and rGOn-PEG Nanosheets

**Figure 3:**
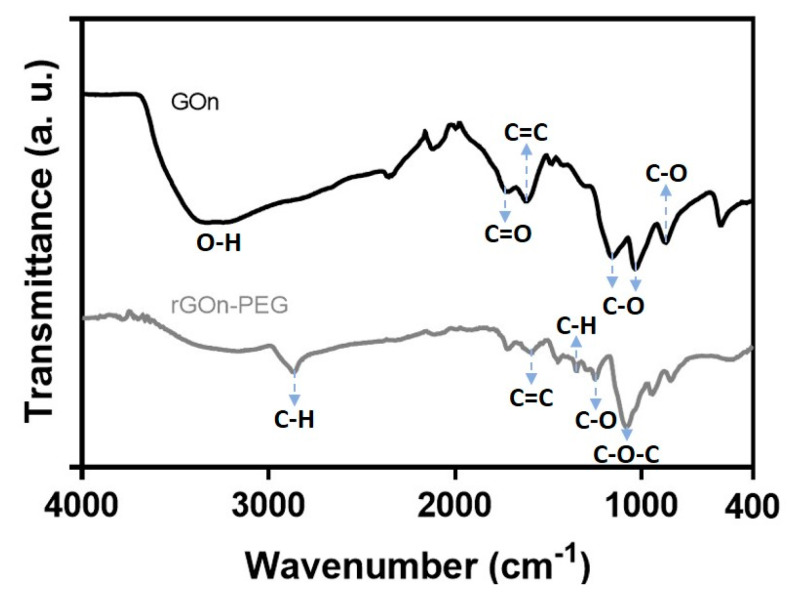
FTIR spectra of GOn and rGOn-PEG. Infrared spectra of GOn (black line) and rGOn-PEG (grey line) describe the contribution of several surface functionalities.

Fourier transform infrared (FTIR) spectra were obtained to confirm the presence of oxygen functionalities on the surface of GOn, as well as its reduction and functionalization into rGOn-PEG (Figure 3). FTIR spectroscopy revealed a broad band in the wavenumber range of 3000 cm^−1^ and 3600 cm^−1^ for GOn, corresponding to O−H stretching vibrations, which are attributed to adsorbed water molecules, hydroxyl, and carboxyl groups [39]. A sharp peak at around 1725 cm^−1^, which is assigned to C=O stretching vibrations, demonstrated the presence of carbonyl and carboxyl groups [30,39]. An absorption band at ≈1616 cm^−1^ appears owing to the stretching of cyclic alkene (C=C) from unoxidized graphitic domain [30,40,41]. Additionally, the presence of ethers is evidenced by the appearance of strong absorption bands at around 1160 cm^−1^ and 1040 cm^−1^, which are assigned to C−O stretching vibrations, and through epoxides exhibiting C−O bending vibrations at around 878 cm^−1^ [39].

The reduction of GOn was demonstrated in FTIR spectra by a decrease on the intensity of peaks corresponding to the oxygen containing functionalities [41], as compared to the intensities of the peaks of graphene oxide (Figure 3). An absorption band at ≈1590 cm^−1^ appears, which is assigned to C=C stretching vibrations, supporting the restoration of π−π structure of the graphitic domain upon reduction. One significant feature from rGOn-PEG IR spectrum is the presence of a strong absorption band at 2876 cm^−1^ and another peak at 1395 cm^−1^. These two bands can be assigned to C−H vibrations, attributed to –CH_2_– or –C–H groups, strongly supporting the adsorption of PEG molecules onto rGOn-PEG [33]. Additionally, the vibrational C−O stretching at 1248 cm^−1^ corresponds to primary alcohols from PEG molecules. The absorption band at 1055 cm^−1^ could be attributed to C−O−C stretching, which is assigned to ether groups of PEG [33].

**Figure 4:**
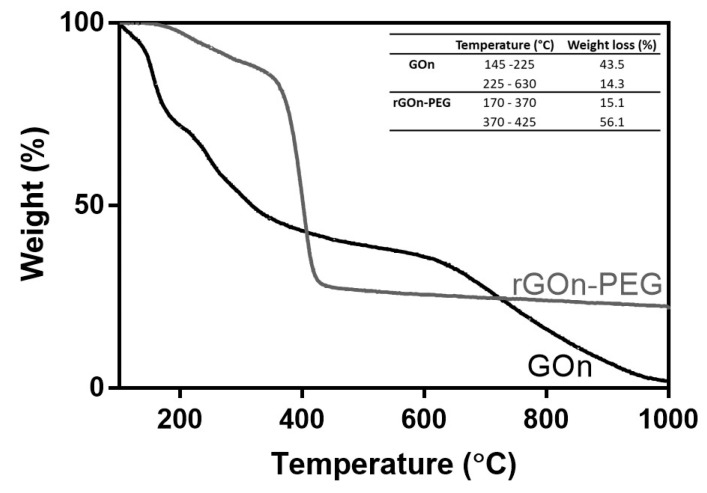
Thermal decomposition of GOn and rGOn-PEG. TGA curves and weight loss values for GOn (black line) and rGOn-PEG (grey line).

Thermogravimetric analysis (TGA) was used to analyze the functionalization degree and thermal stability of GOn and rGOn-PEG. Thermograms for GOn and rGOn-PEG are shown in Figure 4, displaying the weight loss during the heating. The data reveals two main weight loss steps at temperatures above 100 °C for the two materials. The first weight loss occurred between 145 °C and 225 °C for GOn, and between 170 °C and 370 °C for rGOn-PEG. Thermal decomposition during this first step was quantified. It corresponds to the loss of oxygen-containing functional groups, namely carboxyl and epoxy [39]. rGOn-PEG exhibited a lower weight loss when compared with GOn (15.1% and 43.5%, respectively), which suggests fewer oxygen-containing groups are present following reduction and PEGylation [42]. The second weight loss occurred between 225 °C and 630 °C for GOn and 370 °C and 425 °C for rGOn-PEG. In the case of GOn, the weight loss (14.3%) corresponds to the combustion of carbon skeleton and more stable functionalities like carbonyls and residual hydroxyls [43]. The rGOn-PEG substantial weight loss (56.1%) at 425 °C is attributed to the presence of PEG, as at this step pyrolysis of its ether groups occurs [42]. The total percentage weight loss was of 57.8% for GOn and 71.2% for rGOn-PEG. TGA was also performed for non-PEGylated thermally reduced rGOn (Figure S2), which suggests that PEGylation increased the extent of thermal reduction reaction.

**Figure 5:**
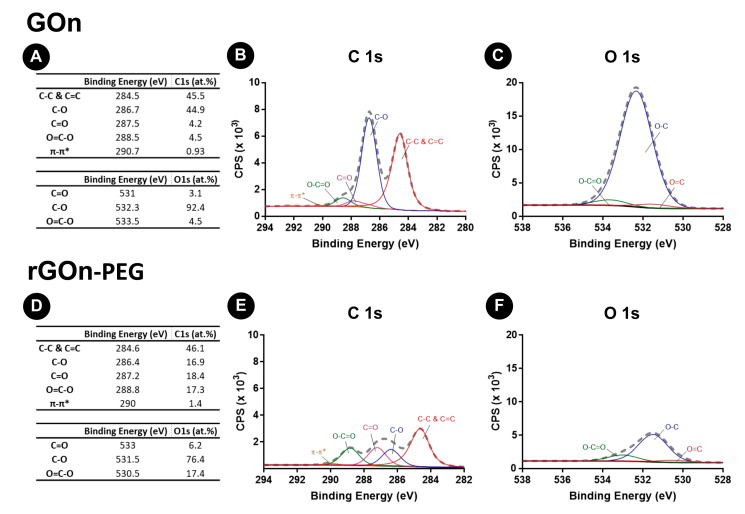
XPS analysis of GOn and rGOn-PEG. (**A**) Atomic composition of GOn and content of C 1s and O 1s chemical functional groups resulting from spectra fitting; (**B**,**C**) Deconvolution of high-resolution (B) C 1s and (C) O 1s XPS spectra for GOn. (**D**) Atomic composition of rGOn-PEG and content of C 1s and O 1s chemical groups resulting from spectra fitting; (**E**,**F**) Deconvolution of high-resolution (E) C 1s and (F) O 1s XPS spectra for rGOn-PEG.

X-ray photoelectron spectroscopy (XPS) analyses were performed to characterize the oxidation degree and chemical functional groups at the surface of GOn and rGOn-PEG (Figure 5).

GOn presented a C *at %* of 62.1 and a O *at %* of 32.0 (Figure S3), which demonstrates a successful oxidation and introduction of oxygen functionalities at its surface. Analysis of C1s spectra of GOn revealed two large peaks, which could be further deconvoluted in five peaks (Figure 5A,B). The first binding energy value was attributed to C−C and C=C (284.5 eV, C1s *at %* = 45.5) due to the formation of *sp^2^* and *sp^3^* hybridizations of carbon in the graphitic backbone. Single bonds of carbon and oxygen (C−O) in hydroxyls are responsible for the second binding energy value (286.7 eV, C1s *at %* = 44.9). This is the most prevalent carbon bond with oxygen. Carbonyl groups are also present in the form of double bonds between carbon and oxygen (C=O, 287.5 eV, C1s *at %* = 4.2%). The occurrence of carboxyls is responsible for the multiple bonds between carbon and oxygen (O=C−O, 288.5 eV, C1s *at %* = 4.5). Finally, it is possible to observe the π–π^*^ bond due to the presence of delocalized π electrons in the graphene lattice (290.7 eV, C1s *at %* = 0.93) [39,44].

Regarding the analysis of O1s spectra of GOn, one peak was observed, which could be deconvoluted in three peaks (Figure 5A,C). The first binding energy value was attributed to C=O due to carbonyl groups (531 eV, O1s *at %* = 3.1), C−O bonds from hydroxyls are responsible for the second binding energy (532.3 eV, O1s *at %* = 92.4) and the third peak can be attributed to carboxyls (O=C−O) (533.3 eV, O1s *at %* = 4.5) [39,45]. The relative abundance of chemical bonds found in both C1s and O1s spectra are in accordance and the analysis of the deconvoluted spectra showed that GOn was well oxidized due to the presence of carbon atoms in functional groups (hydroxyl, carbonyl, and carboxyl) with C−O bonds dominating the surface chemistry (Figure 5A).

rGOn-PEG presented a C *at %* of 63.5%, an O *at %* of 15.9%, and a N *at %* of 20.6% (Figure S3). These results demonstrated a decrease of the oxygen content comparing with GOn and the presence of N, resulting from the amine-terminated groups from PEG-NH_2_. Analysis of C1s and O1s spectra was also performed (Figure 5D–F). Similarly to GOn, C1s spectra was deconvoluted in five peaks. However, different relative abundances in oxygen containing functionalities were found (Figure 5E). The C−C and C=C bond (284.6 eV) continued to largely exist, accounting for 46.1% of carbon bonds (Figure 5D,E). C−O bond (286.4 eV), the most abundant oxygen functionality in GOn, decreased significantly to 16.9% after chemical modification into rGOn-PEG (Figure 5D). The impact of reduction on surface chemistry was also shown by a large increase of C=O bond (287.2 eV) to 18.4% and through the development of a strong peak of O=C−O bond (288.8 eV) with 17.3% of the carbon bonds in rGOn-PEG. In terms of π–π^*^ bond (290 eV), the results indicate a possible restoration of aromatic structure in rGOn-PEG with an increase from 0.9% in GOn to 1.4% in rGOn-PEG [46,47]. In the case of O1s spectra, it was deconvoluted in three peaks (Figure 5F). An increase of C=O (533 eV, O1s *at %* = 6.2) and O=C−O bonds (530.5 eV, O1s *at %* =17.4) was observed, while there was a decrease on the content of C−O bonds (531.5 eV, O1s *at %* =76.4). These results corroborate those obtained for the C1s spectra. Also, C/O ratio for GOn and rGOn-PEG was of 1.9 and 4, respectively, indicating that rGOn-PEG was successfully reduced. XPS spectra were also obtained for non-PEGylated thermally reduced rGOn (Figure S4), showing a lower extent of reduction in comparison to rGOn-PEG. Successful PEG-NH_2_ surface adsorption in rGOn-PEG was confirmed by the presence of a N *at %* of 20.62 (deconvoluted N1s spectra, Figure S5).

### 3.3. Optical Properties and Photothermal Effect

**Figure 6:**
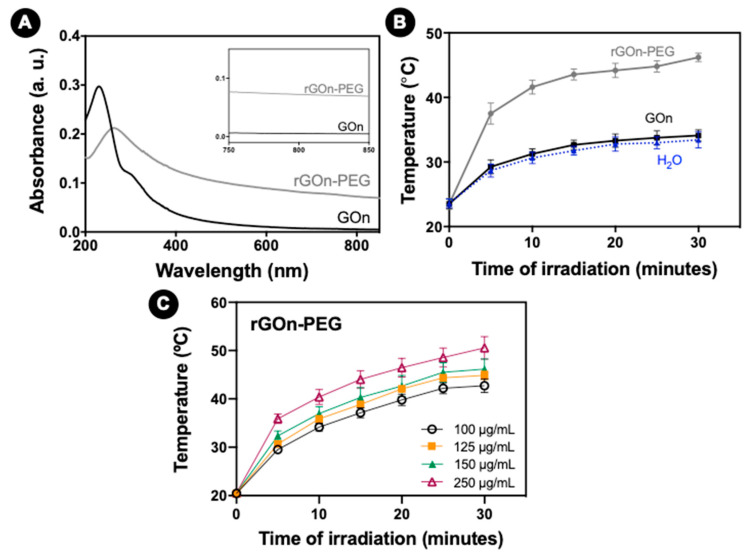
NIR absorption capacity of GBM. (**A**) UV/vis absorption curves of GOn and rGOn-PEG. The inset shows a zoom-in view of the curves in the NIR range from 750 nm to 850 nm. (**B**) Photothermal heating curves of water (dashed line, blue), and GOn (black) and rGOn-PEG (grey) aqueous dispersions at a concentration of 250 μg mL^−1^ in water. (**C**) Concentration-dependent effect on photothermal heating curves for rGOn-PEG.

To assess the potential of the developed materials to be used for photothermal therapy, namely in terms of NIR absorption capacity, the optical properties of both GOn and rGOn-PEG were determined by UV/vis spectroscopy (Figure 6A). Absorbance measurements showed an absorbance peak at *λ*_max_ = 230 nm for GOn, which is assigned to π–π^*^ electronic transitions in *sp*^2^ clusters, and a shoulder peak at 300 nm, which is attributed to n–π^*^ transitions of free electron pairs in oxygen atoms in C=O bonds from carbonyl and carboxyl groups [39]. A red shift of *λ*_max_ to 263 nm was observed for rGOn-PEG. Additionally, rGOn-PEG exhibited ≈13-fold increment over GOn in NIR absorbance (at 810 nm, Figure 6A inset). These results also support that thermal reduction and non-covalent PEGylation strongly reduced GOn.

To further investigate the ability of these nanomaterials to convert NIR light energy into thermal energy, heat generation upon NIR irradiation was evaluated for rGOn-PEG, GOn and water only as control. As seen in Figure 6B, upon 30 min of NIR irradiation, GOn remained at 34 °C, comparable to water. rGOn-PEG displayed concentration-dependent photothermal heating (Figure 6C). In comparison with GOn, aqueous solutions containing 250 μg mL^−1^ of rGOn-PEG showed a higher temperature increase (≈42 °C and ≈47 °C, after 10 and 30 min of NIR irradiation, respectively).

### 3.4. In Vitro Biocompatibility of GBM Dispersions

**Figure 7:**
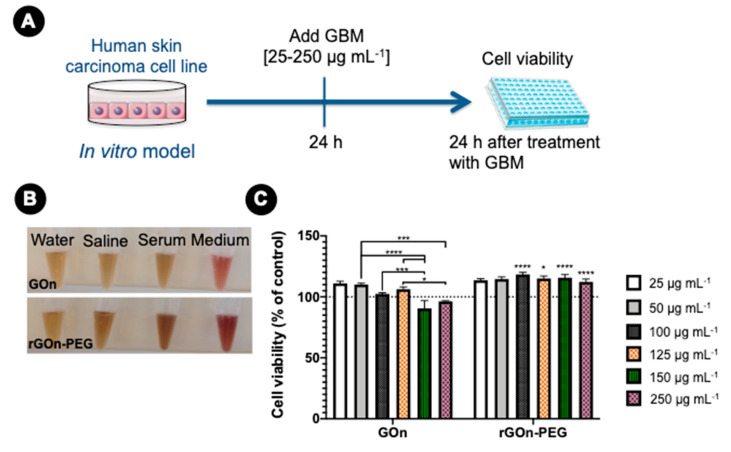
GBM biocompatibility. (**A**) Experimental set-up. One day (24 h) after seeding, human skin carcinoma cells (A-431) were treated with different concentrations of GOn and rGOn-PEG and incubated for an additional 24-h period, prior to resazurin assay. (**B**) GOn and rGOn-PEG (250 μg mL^−1^) in water and physiological solutions. (**C**) Cellular viability determined using resazurin assay. Results are normalized with respect to values of the control without GBM. Statistically significant differences are shown as * *p* < 0.05, *** *p* < 0.001, **** *p* < 0.0001. Dashed line represents 100% cell viability of the control without GBM.

To evaluate the biological effect of GOn and rGOn-PEG alone, A-431 cells were used as in vitro model of human skin carcinoma (Figure 7A). Firstly, the dispersibility of GOn and rGOn-PEG in different physiological solutions (PBS, FBS, and complete culture medium) was macroscopically monitored and no precipitates were observed in any of the dispersions up to the highest concentration tested (250 μg mL^−1^, Figure 7B). Then, cells were incubated with increasing concentrations of developed GBM for 24 h followed by cell viability assessment through resazurin assay (Figure 7A). As control, A-431 cells were cultured in the absence of any material. Increasing concentrations of GOn and rGOn-PEG (25, 50, 100, 125, 150, and 250 μg mL^−1^) did not affect cell viability, as compared to controls (Figure 7C). Nonetheless, a tendency for a reduction in cell viability was observed for higher concentrations of GOn (150 and 250 μg mL^−1^, *p* < 0.05). In opposition, rGOn-PEG elicited viability levels above those of control condition. Altogether, these results showed that both GOn and rGOn-PEG do not induce harmful effects on A-431 cells at the concentrations tested, but PEGylation might improve in vitro biocompatibility of these 2D nanomaterials.

### 3.5. In Vitro Photothermal Effect of rGOn-PEG

**Figure 8:**
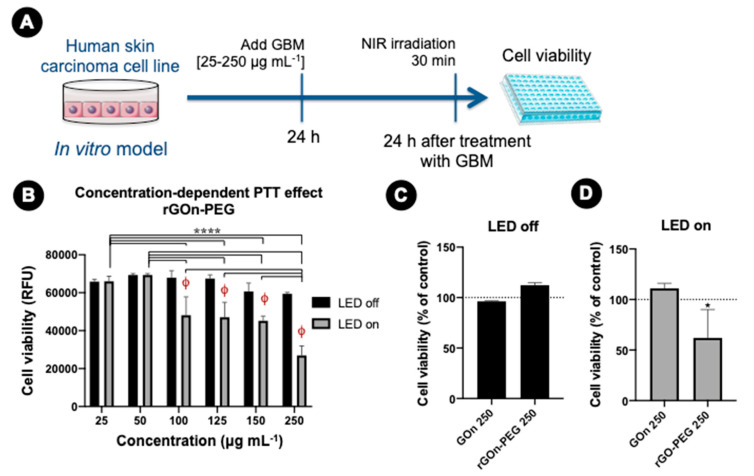
NIR irradiation and in vitro photothermal effect. (**A**) Experimental set-up. One day (24 h) after seeding, human skin carcinoma cells (A-431) were treated with different concentrations of GOn and rGOn-PEG and incubated for an additional 24-h period, prior to NIR irradiation for 30 min and resazurin assay. (**B**) Cellular viability determined using resazurin assay. Results are shown as relative fluorescence units (RFU). Statistically significant differences are shown as ****, *p* < 0.0001, φ, *p* < 0.0001 in comparison to LED off condition. (**C**) Cellular viability without NIR irradiation in the presence of 250 μg mL^−1^ of GOn or rGO-PEG. Results are normalized with respect to values of the control without GBM (**D**) Cellular viability upon NIR irradiation in the presence of 250 μg mL^−1^ of GOn or rGOn-PEG. Results are normalized with respect to values of the control without GBM. Statistically significant differences are shown as *, *p* < 0.05.

To determine the cytotoxicity of GOn and rGOn-PEG under NIR irradiation, A-431 cells were incubated with GBM dispersions as described above and then irradiated with a 810 nm LED source for 30 min, followed by cell viability assay (Figure 8A). Increasing concentrations of rGOn-PEG induced a significant decrease in cell viability upon NIR irradiation. Particularly, A-431 cells incubated with > 100 μg mL^−1^ of rGOn-PEG showed significantly lower values in the resazurin assay, in comparison to their non-irradiated counterparts (*p* < 0.0001, Figure 8B,C). Indeed, irradiation of A-431 cells incubated with 250 μg mL^−1^ of rGOn-PEG induced an approximate 38% decrease of cell viability (*p* < 0.05, Figure 8D). These results suggest a potential PTT effect of rGOn-PEG upon low-power (150 mW cm^−2^) NIR irradiation. As control, no PTT effect was observed for cells incubated with 250 μg mL^−1^ of GOn upon NIR irradiation (Figure 8D).

## 4. Discussion

Graphene-based materials have been increasingly investigated for applications in nanomedicine, particularly as PTT platforms to improve the efficacy of cancer treatment strategies. The 2D nanomaterial graphene oxide is commonly obtained from graphite by exfoliation methods, like the modified Hummer’s method used here and in our previous work [27]. The exfoliation process results in the introduction of several oxygen functionalities and consequent loss of structural, thermal, and electrical properties [24,28]. In order to restore some of the properties of pristine graphene, over the years different reduction methods have been explored to produce reduced graphene oxide (rGO), including chemical, thermal, and electrochemical reduction pathways [24]. However, the reduction of oxygen functional groups at the surface of GO leads to the formation of unstable colloidal dispersions in aqueous solutions, limiting their potential for biomedical applications and requiring further chemical functionalization toward improving water solubility [25]. Covalent and non-covalent functionalization of GO and rGO with biocompatible polymers like PEG has been widely reported [25,30,31,33,48], using different methods and polymer characteristics (molecular weight, chemistry, etc.). Single step reduction and PEGylation of graphene oxide has been previously described by Chen et al. using a water bath at 90 °C for 24 h and methoxypolyethylene glycol amine (mPEG-NH_2_, *M*_n_ = 5 kDa) [37]. The authors reported improved water stability of the PEGylated reduced graphene oxide and increased release of resveratrol upon irradiation using a high power 808 nm laser. Following this protocol, we used polyethylene glycol bis(amine) to prepare PEGylated reduced graphene oxide with small sizes for low power NIR-light triggered PTT applications, using cheaper, smaller, and safer LEDs. For this purpose, nano-sized GO (GOn) was obtained by ultrasonication of GO, rendering nanoplatelets with average lateral dimensions below 300 nm. As previously demonstrated, the sonication step has a strong impact on the size of GO flakes, without considerably changing other physicochemical properties [39]. Purified GOn dispersions with controlled lateral dimensions were then reduced and non-covalently functionalized with PEG to attain stable aqueous dispersions with high NIR absorption capacity. The chemical signature of obtained rGOn-PEG colloidal dispersions was carefully investigated through multiple complementary techniques to assess the impact of one-step reduction and PEGylation on the physicochemical properties of graphene oxide.

Upon chemical modification, a significant increase in lateral dimensions from ≈287 nm to ≈521 nm, was found, suggesting the attachment of PEG molecules to GOn. Such an increase in size after PEGylation has been reported by others [33,49]. Smaller sizes have been reported by introducing a sonication step during PEG conjugation reaction [25]. It is relevant to notice that nano-sized particles have been reported to result in improved skin permeation and skin retention, improving treatment outcomes of inflammatory skin diseases [35,36].

The introduction of oxygen functionalities on the surface of GOn, as well as the reduction and non-covalent functionalization of rGOn-PEG were confirmed by FTIR, TGA, and XPS. In comparison with GOn, rGOn-PEG exhibited lower oxygen content given that the majority of oxygen-containing functionalities (carboxyl, hydroxyl, and ketone groups) were removed during the reduction process. Chen and colleagues have previously reported that covalent PEGylation of GOn preserved the aromatic structure of GOn and that a similar green reduction protocol (24 h at 90 °C in a water bath) was able to recover the aromatic structure on rGO-PEG by repairing defects caused during the removal of oxygen moieties from GOn [32]. Additionally, comparing with rGO, simultaneous functionalization with PEG was demonstrated, not only to improve water solubility, but also to increase the extent of the reduction reaction, as confirmed by complementary techniques (TGA and XPS). Other studies have also attributed a role to PEG in strengthening the reduction extent of GOn [32,37]. Indeed, amine groups, which can be oxidized to nitrite, have been reported to exhibit mild reductive ability, being employed as reducing reagents in the preparation of rGO [29]. Similarly to PEG-NH_2_, gelatin has numerous amine groups in its backbone and has been reported to act as a reducing agent in the production of stable reduced graphene oxide nanosheets under mild heating conditions [29,50]. In these cases, gelatin formed covalent bonding with rGO through its amine groups. The chemical reactivity of graphene oxide toward amines has been explained by different routes in the literature. Particularly, different types of amine can react with GO functional groups via amidation reaction of carboxylic acid groups at the edges of GO or through ring-opening of epoxides on the surface of GO [50,51,52]. Hydrogen bonding between amines and hydroxyls of GO is another possibility [32,53]. Nonetheless, given the complex structure of graphene-based materials, the nature of such chemical reactions is still far from being fully understood.

According to TGA measurements, the ratio of grafted PEG was estimated to be 56.1%, supporting the efficiency of single step reduction and PEGylation. Previous reports on rGOn-PEG prepared through a similar method does not provide quantitative data regarding the chemical modification of the material [37], but other non-covalent methods have shown around 78% modification through PEGylation of previously reduced GO [33]. It is worth noting that such non-covalent functionalization methods rely on polymer physisorption onto graphene basal planes via π–π stacking and van der Waals interactions and few examples of covalent functionalization of rGO exist owing to the removal of the majority of reactive functional groups upon reduction [24]. Notwithstanding, non-covalent functionalization methods exhibit strong advantages over covalent functionalization, including the preservation of extended π conjugation and the aromatic structure of GOn, whereas covalent modification creates sp^3^ defects on the graphene ring [54].

PEGylation was successful in terms of overcoming the hydrophobicity associated to rGO and rendering a nanomaterial that is stable in different physiological media (PBS, FBS, and culture medium). Herein, we demonstrated that rGOn-PEG had largely restored its aromatic structure showing strongly enhanced absorbance in the NIR region (≈13-fold increase, in comparison to GOn) and light-to-heat energy conversion capacity upon NIR irradiation, comparable to the material previously obtained using a similar protocol with a different PEG [37]. On the other hand, other studies reported approximately 6- to 8-fold increment in NIR absorption by rGOn-PEG produced through covalent PEGylation followed by thermal reduction [32,49]. Several studies have shown a very rapid heating of graphene-based nanomaterials using more powerful irradiation systems (>3 W cm^−2^, compared to 150 mW cm^−2^ used herein) and reporting heating up to 60–70 °C [32,37,55,56]. Such high temperatures are above those needed for tumor ablation treatments (> 50 °C) [7], and are also likely to induce damage in healthy cells of surrounding tissues, particularly considering the small sizes of superficial non-melanoma skin cancers, such as basal cell carcinoma (≈20 mm in size). For instance, protein-functionalized rGO nanosheets (40 μg mL^−1^) and polyethylenimine-PEG-rGO (8 μg mL^−1^) induced a reduction of 35% in cell viability without irradiation [55,57]. Herein, we demonstrated that both GOn and rGOn-PEG alone and below 250 μg mL^−1^ were noncyotoxic to A-431 epidermoid carcinoma cells. PEGylation is an effective chemical functionalization commonly used to improve the biocompatibility of nanomaterials [25,30,34]. Although a tendency for a decrease in cell viability was observed with increasing concentrations of unmodified GOn, the same was not observed for rGOn-PEG, for which concentrations > 100 μg mL^−1^ resulted in higher fluorescence measurements using resazurin assay, in comparison to control. This result supports the use of biocompatible polymers as PEG to enhance the biological effects of developed nanomaterials.

To further determine the in vitro photothermal effect of rGOn-PEG, we irradiated A-431 cells in the presence of prepared GBM using low power (150 mW cm^−2^) NIR LEDs. Consistently with other studies [32,49,58], NIR irradiation alone was not sufficient to induce cell death, but the combination of rGOn-PEG with NIR irradiation resulted in ≈38% decrease of cell viability after a single treatment during 30 min. As described above, rGOn-PEG temperature increased up to 47 °C upon NIR irradiation, which falls within the hyperthermia range of temperatures (41–50 °C) [6,7]. Hyperthermia triggers apoptotic pathways, interfering with normal cell functions, possibly leading to enhanced membrane permeability, metabolic signaling disruption, dysfunctional membrane transport, and activation of heat shock proteins, among other cellular and molecular changes [7,59,60]. Nonetheless, the effects of hyperthermia, and particularly their combination with graphene-based nanomaterials, are still far from being fully understood. To the best of our knowledge, our study is the first to report the effect of reduced graphene oxide on epidermoid carcinoma cells, which are commonly used as in vitro models of non-melanoma skin cancer. The effect of nanocomposites of gold nanorod-assembled PEGylated graphene oxide has been reported to result in similar levels of cytotoxicity (≈40% decrease in cell viability) upon irradiation with high power (60 W cm^−2^) Xe-lamp light [58]. Others have shown that comparable PEGylated graphene-based nanomaterials elicited ≈80% cell viability decrease using 4T1 breast cancer cell line [37]; and up to ≈90% decrease of the viability of A549 adenocarcinomic human alveolar basal epithelial cells using covalent chemical functionalization methods [32]. Notwithstanding, major differences in the irradiation time and power of the light source limit the comparison between studies.

## 5. Conclusions

In this work, we characterized the impact of a single step thermal reduction and PEGylation process on the physicochemical properties of graphene oxide using multiple complementary techniques. Chemical modification with PEG not only resulted in improved water dispersibility of reduced graphene oxide, but also contributed to enhance the extent of the thermal reduction reaction. PEGylation yielded single-layer rGOn-PEG sheets with average nano-sized lateral dimensions of ≈521 nm. The single step process resulted in the restoration of the aromatic structure of graphene, evidenced by the appearance of C=C bonds in rGOn-PEG infrared spectrum, increased C/O ratio, and changes in the optical properties through a red shift of *λ*_max_. The obtained rGOn-PEG exhibited a ≈13-fold increase in NIR absorbance and reached 42 °C after 10 min of NIR LED irradiation, exhibiting a continuous heating up to 47 °C after 30 min, whereas unmodified GOn remained at 34 °C even under NIR irradiation. Temperatures registered for rGOn-PEG were within the hyperthermia range. PEGylation of rGOn resulted in improved in vitro biocompatibility, compared to unmodified GOn, which seemed to induce a reduction of A-431 cell viability for concentrations above 150 μg mL^−1^. Combining NIR irradiation with rGOn-PEG in concentrations above 100 μg.mL^−1^ resulted in a cytotoxic effect. After a single irradiation with a low power NIR LED system, a 38% decrease of cell viability was found, showing the in vitro photothermal effect of rGOn-PEG.

Altogether, our results further support the use of a simple and facile method to obtain functionalized rGOn as a promising photoabsorbing agent for PTT applications in non-melanoma skin cancer treatment. The combination of this functionalized nanomaterial with NIR irradiation using a safer LED-based NIR light source opens new possibilities toward exploring lower power and cheaper systems for mild hyperthermia cancer therapy, enabling better control over nanomaterial heating.

## Figures and Tables

**Table 1:** Surface charge of GOn and rGOn-PEG aqueous dispersions at an initial concentration of 50 μg mL^−1^ and pH 7.4 (*n* = 3)

GBM	Surface Charge (mV)
GOn	−25.1 ± 0.8
rGOn-PEG	−10.2 ± 0.3

## Supplementary Materials

The following are available online at https://www.mdpi.com/2073-4360/12/8/1840/s1. Figure S1. Volume distribution of particle size of GOn and rGOn-PEG dispersed in water at an initial concentration of 250 μg mL^-1^ and determined by light scattering using a Coulter counter. A boxplot, which is a standardized way of displaying the dataset based on a five-number summary (the minimum, the maximum, the sample median, and the first and third quartiles) is presented in Figure 2C. Figure S2. TGA curve of rGOn and weight loss. Figure S3. XPS survey spectra and atomic composition for (A) GOn and (B) rGOn-PEG. Figure S4. Surface chemical properties of rGOn. (A) Atomic composition of rGOn and content of C 1s and O 1s chemical groups resulting from spectra fitting; (B, C) Deconvolution of high-resolution (B) C 1s and (C) O 1s XPS spectra. Figure S5. Quantification of functionalization of rGOn-PEG. (A) Content of N 1s chemical groups resulting from spectra fitting; (B) Deconvolution of high-resolution N 1s XPS spectra.
