# Supplementary material for: Near-Infrared Radiation-Based Mild Photohyperthermia Therapy of Non-Melanoma Skin Cancer with PEGylated Reduced Nanographene Oxide
Source: Polymers (Basel). 2020 Aug 17;12(8):1840. doi: 10.3390/polym12081840 (PMC7466052; doi:10.3390/polym12081840)
Supplement: Supplementary file 1 [file polymers-12-01840-s001.pdf]

SUPPORTING INFORMATION

Near-Infrared Radiation-Based Mild Photohyperthermia Therapy of Non-Melanoma Skin Cancer with PEGylated Reduced Nanographene Oxide

Raquel Costa-Almeida [1,2,†], Diana Bogas [3,†], José R. Fernandes [4], Licínia Timochenco [3], Filipa A. L. S. Silva [1,2], João Meneses [3], Inês C. Gonçalves [1,2], Fernão D. Magalhães [3] and Artur M. Pinto [1,2,3,*]

[1] i3S—Instituto de Investigação e Inovação em Saúde, Universidade do Porto, Porto 4200-180, Portugal; rcalmeida@i3s.up.pt (R.C.-A.); flsilva@i3s.up.pt (F.A.L.S.S.) icastro@ineb.up.pt (I.C.G.)

[2] INEB—Instituto de Engenharia Biomédica, Universidade do Porto, Rua Alfredo Allen, 208, Porto 4200-180, Portugal

[3] LEPABE, Faculdade de Engenharia, Universidade do Porto, Porto 4200-180, Portugal; dianabogas@fe.up.pt (D.B.); up201809122@fe.up.pt (L.T.); up201503132@fe.up.pt (J.M.); fdmagalh@fe.up.pt (F.D.M.)

[4] CQVR—Centro de Química Vila Real, Departamento de Física, ECT, Universidade de Trás-os-Montes e Alto Douro, Vila Real, Portugal 5001-801; jraf@utad.pt

* Correspondence: arturp@fe.up.pt

† These author contributed equally to this work.

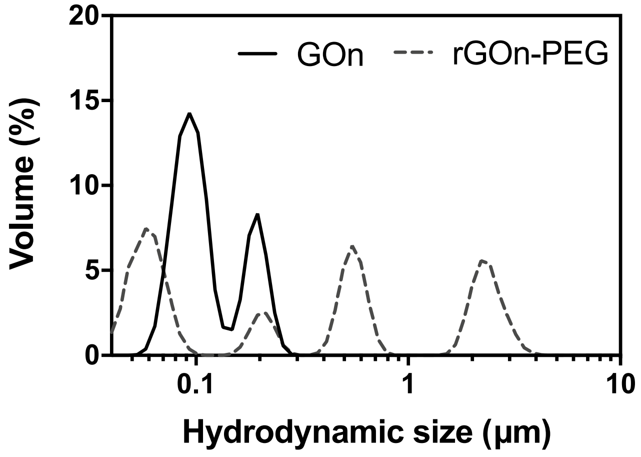

Figure S1. Volume distribution of particle size of GOn and rGOn-PEG dispersed in water at an initial concentration of 250 μg mL^{-1} and determined by light scattering using a Coulter counter. A boxplot, which is a standardized way of displaying the dataset based on a five-number summary (the minimum, the maximum, the sample median, and the first and third quartiles) is presented in Figure 2C.

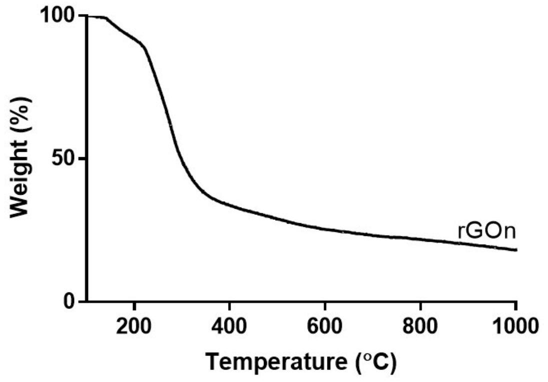

Figure S2. TGA curve of rGOn and weight loss.

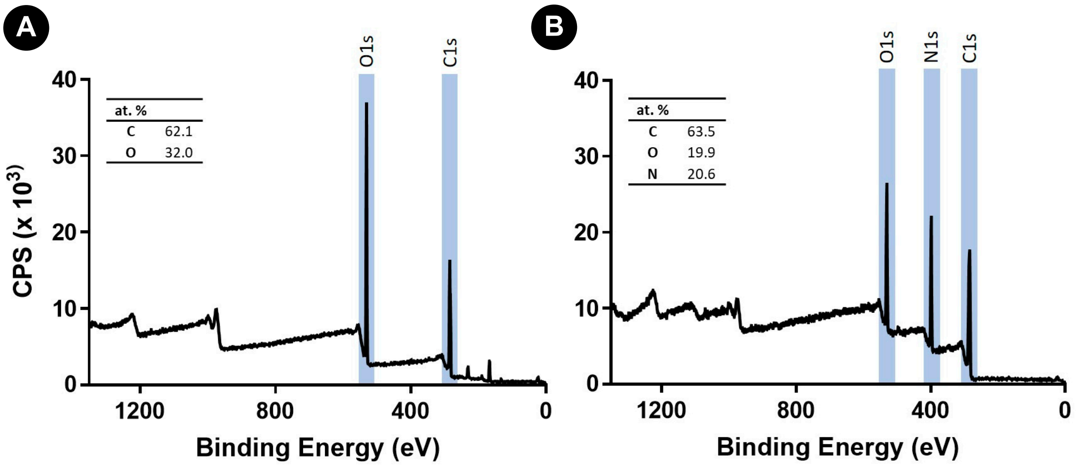

Figure S3. XPS survey spectra and atomic composition for (**A**) GOn and (**B**) rGOn-PEG.

A

	Binding Energy (eV)	C1s (at.%)
C-C & C=C	284.6	27.3
C-O	286.2	59.2
C=O	287.1	9.7
O=C-O	288.6	3
π-π*	290	0.8

	Binding Energy (eV)	O1s (at.%)
C=O	531	9.1
C-O	532.5	84.3
O=C-O	533.8	6.6

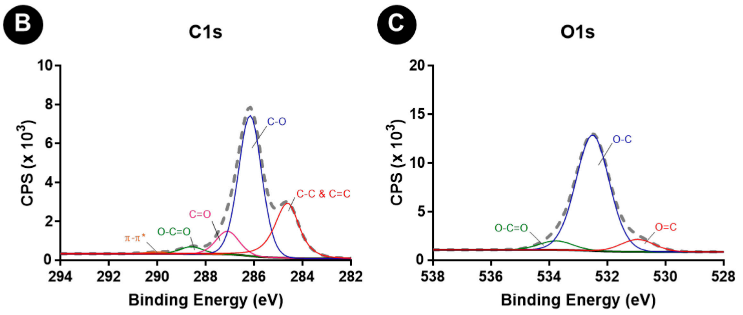

Figure S4. Surface chemical properties of rGOn. (**A**) Atomic composition of rGOn and content of C 1s and O 1s chemical groups resulting from spectra fitting; (**B**, **C**) Deconvolution of high-resolution (B) C 1s and (C) O 1s XPS spectra.

A

	Binding Energy (eV)	N1s (at.%)
NH_2	399.7	93.1
NH-C=O	400.9	6.9

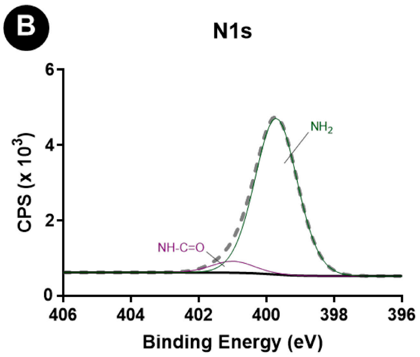

Figure S5. Quantification of functionalization of rGOn-PEG. (**A**) Content of N 1s chemical groups resulting from spectra fitting; (**B**) Deconvolution of high-resolution N 1s XPS spectra.
